# Frost Resistance and Mechanism of Circulating Fluidized Bed Fly Ash-Blast Furnace Slag-Red Mud-Clinker Based Cementitious Materials

**DOI:** 10.3390/ma15186311

**Published:** 2022-09-12

**Authors:** Wei Zhang, Chao Wei, Xiaoming Liu, Zengqi Zhang

**Affiliations:** 1 State Key Laboratory of Advanced Metallurgy, University of Science and Technology Beijing, Beijing 100083, China; 2 School of Metallurgical and Ecological Engineering, University of Science and Technology Beijing, Beijing 100083, China

**Keywords:** circulating fluidized bed fly ash, frost resistance, pore structure, harmful elements, cementitious materials

## Abstract

The motivation of this work is to enhance the long-term frost resistance of circulating fluidized bed fly ash (CFA)-based multisolid waste cementitious material (CSM). In this research, CSM2 is prepared by 30 wt.% CFA, 20 wt.% blast furnace slag (BFS), 10 wt.% red mud (RM), 10 wt.% phosphorus slag (PS), and 30 wt.% cement clinker (CC). The strength and mass of CSM are detected by a press and electronic balance. The hydration products, polymerization degree, thermogravimetric, micromorphology, pore structure, and harmful element leaching are detected by XRD, MAS NMR, TG-DTG, SEM-EDX, MIP, and ICP-MS. The major findings indicate that the strength loss, mass loss, and strength of CSM2 after 25 freeze–thaw cycles (CSM2-25) are 2.35%, 0.36%, and 49.95 MPa, respectively, which is superior to other CSMs and still meets the performance requirements of fly ash Portland cement 42.5#. The main hydration products are C-S-H gel, C/N-A-S-H gel, and ettringite during the freeze–thaw cycle. The polymerization degree and thermogravimetric loss of hydration products in CSM2-25 are 50.65% and 12.82 wt.%, respectively, which are higher than those of other CSMs under the synergy of CFA, BFS, RM, and PS. In addition, the microscopic results show that the interface between the paste and aggregate, micromorphology, and pore structure of CSM2-25 are the densest when the mass ratio of Ca/(Si + Al) is 0.81. These characteristics are beneficial to the improvement of long-term frost resistance in CSM2. Finally, the leaching results of harmful elements in CSM2 after 25 freeze–thaw cycles still meet the WHO standard of drinking water. Therefore, this work provides a reliable reference for the preparation of green cementitious materials with great frost resistance by using CFA, BFS, RM, and PS.

## 1. Introduction

The frost resistance of concrete refers to its ability to maintain strength and appearance integrity under the action of freeze–thaw cycles, which is one of the important indicators of concrete durability [1,2]. In addition, internal stress and cracks are generated in the freeze–thaw cycle process of concrete [3]. At the same time, the performance and internal structure of concrete are destroyed. Specifically, the internal cracks of concrete are increased due to the repeated effect of seasonal temperature changes, which lead to the strength loss and mass loss of concrete [4,5]. Concrete with poor frost resistance cannot reach normal service life, resulting in rework, transformation, and other investment waste [6,7]. Therefore, the frost resistance of concrete is very important for the development of the construction industry.

Concrete is one of the most important building engineering materials, which is usually prepared by cement and aggregate [8,9]. However, the production of traditional cement not only consumes energy and natural materials but also discharges a large amount of CO_2_ [10,11]. According to the statistics of relevant departments, production of cement in China was 2.36 billion tons in 2021, which is approximately 55% of the cement production in the world. The CO_2_ emissions of the cement industry in China are 1.36 billion tons in 2021, accounting for 12% of the total CO_2_ emissions of the country. Therefore, the cement industry is the key industry for CO_2_ emissions in China. The reduction task of CO_2_ emissions from cement is arduous and faces new opportunities for development. At present, it has become a developing trend to utilize solid waste as a partial replacement for cement in the preparation of low-carbon cementitious materials [12,13,14]. This method is not only beneficial to reduce CO_2_ emissions in the cement industry and environmental pollution of solid waste but also to improve the utilization of solid waste [15,16]. Therefore, the preparation of cementitious materials using solid waste is a research hotspot in the construction industry to reduce cement consumption [17,18].

Circulating fluidized bed fly ash (CFA) and red mud (RM) are the byproducts of power plants and aluminum plants in China, respectively [19,20,21]. The annual average emissions of CFA and RM are 280 million tons and 105 million tons, respectively, and their harmful elements can pollute the ecological environment [22,23,24]. Relevant scholars found that the excellent strength, volume stability, and environmental performance were reflected when the cementitious material was prepared by CFA and RM at hydration for 28 days [23]. Blast furnace slag (BFS) and phosphorus slag (PS) are solid wastes from ironmaking plants and phosphorus plants that contain active silicon-aluminum components [25,26]. Its mechanical properties, micro densification, and polymerization degree are improved at curing for 28 days as the cementitious material contains BFS and PS [27,28]. The above research has contributed much to the utilization of CFA, RM, BFS, and PS to improve the performance of cementitious materials at 28 days. However, there are few studies that have focused on the long-term (>28 days) frost resistance of cementitious materials prepared from CFA, RM, BFS, and PS. In particular, the frost resistance mechanism of the cementitious materials is missing. Therefore, an effective scheme is proposed by this study to fill this research gap.

In this research, circulating fluidized bed fly ash-based multisolid waste cementitious material (CSM) is prepared by CFA, BFS, RM, and a silicon-aluminum-based solid waste (fine blast furnace slag (FBFS)/PS/gasification slag (GS)). The freeze–thaw cycle experiment of CSM is investigated to understand the frost resistance. Concretely, the frost resistance of the three CSMs are comprehensively compared, and the optimal mass ratio of Ca/(Si + Al) is determined according to the frost resistance in CSM. More importantly, the mechanism of frost resistance in CSM has been discussed in detail during 25 freeze–thaw cycles. Therefore, this work is expected to provide a novel idea to create the long-term frost resistance of cementitious materials by using the synergy of CFA, BFS, RM, and PS.

## 2. Materials and Methods

### 2.1. Physicochemical Performances of Raw Materials

#### 2.1.1. Raw Materials

The circulating fluidized bed fly ash (CFA)-based multisolid waste cementitious material (CSM) is composed from CFA, Bayer red mud (RM), blast furnace slag (BFS), cement clinker (CC), and a silicon-aluminum-based solid waste (fine blast furnace slag (FBFS)/phosphorus slag (PS)/gasification slag (GS)). CFA and RM are provided by a thermal power plant and an aluminum plant, respectively, in Yangquan City, Shanxi Province, China. BFS is supplied by Longze Co., Ltd., Gongyi City, Henan Province, China. CC is produced from a cement plant in Hebei Province, China. Silicon-aluminum-based solid waste: FBFS comes from a steel plant in Hejin City, Shanxi Province, China. PS and GS are provided from a phosphorus plant in Guizhou, China and a coal gasification plant in Inner Mongolia, China, respectively.

#### 2.1.2. Chemical Compositions

The oxide compositions of the CFA, RM, BFS, CC, and silicon-aluminum-based solid waste (FBFS, PS and GS) are shown in Table 1. The main chemical components of CFA are T-CaO (CaO), SiO_2_, Al_2_O_3_, SO_3_, Fe_2_O_3_, and f-CaO. The chemical components of RM are CaO, SiO_2_, Al_2_O_3_, Fe_2_O_3_, and Na_2_O. The main oxides of BFS, FBFS, PS, and GS include CaO, SiO_2_, and Al_2_O_3_. CC is one of the raw materials for preparing cement, and its chemical components are CaO, SiO_2_, and Al_2_O_3_.

#### 2.1.3. Phase Composition

**Figure 1:**
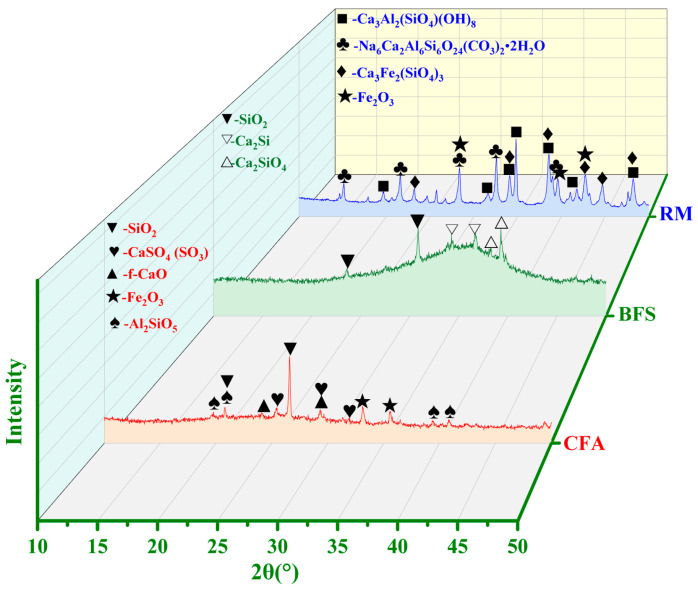
XRD results of RM, BFS, and CFA.

The mineral composition of CFA, RM, and BFS is shown in Figure 1. The main minerals of CFA are active silicon aluminum, quartz (SiO_2_), anhydrite (CaSO_4_ (SO_3_)), hematite (Fe_2_O_3_), free calcium oxide (f-CaO), and kyanite (Al_2_SiO_5_). The phases of RM are composed by katoite (Ca_3_Al_2_ (SiO_4_) (OH)_8_), cancrinite (Na_6_Ca_2_Al_6_Si_6_O_24_ (CO_3_)_2_ 2H_2_O), andradite (Ca_3_Fe_2_ (SiO_4_)_3_), and hematite (Fe_2_O_3_). The main phases of BFS are the active silicon aluminum, calcium silicon (Ca_2_Si), dicalcium silicate (Ca_2_SiO_4_), and SiO_2_. 

**Figure 2:**
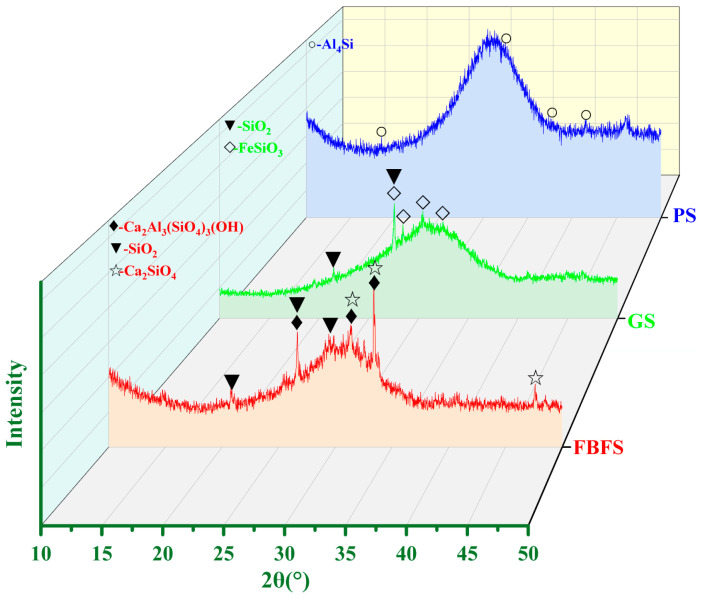
XRD results of FBFS, PS, and GS.

Figure 2 shows the XRD results of silicon-aluminum-based solid wastes (PS, GS, and FBFS). The phase of PS is composed by active Si-Al and aluminum silicon (Al_4_Si). The mineral composition of GS includes active Si-Al, SiO_2_, and clinoferrosilite (FeSiO_3_). The main minerals of FBFS are active Si-Al, quartz (SiO_2_), calcium silicate (Ca_2_SiO_4_), and zoisite (Ca_2_Al_3_ (SiO_4_)_3_ (OH)). These active Si-Al participates in the secondary hydration reaction to generate gel products, which are for the development of long-term frost resistance in the CSM system rather than FBFS and GS.

#### 2.1.4. Specific Surface Area

**Figure 3:**
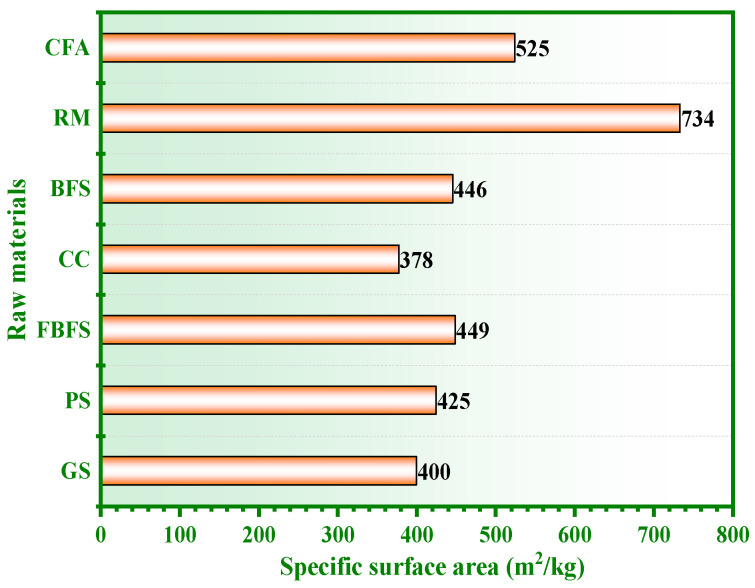
Specific surface area in CFA, RM, BFS, CC, FBFS, PS, and GS.

The particle size of solid wastes and cementitious materials is usually expressed by the specific surface area. CFA, RM, BFS, CC, FBFS, PS, and GS were ground in a cement mill for a certain time. Then, the specific surface areas of the raw materials were tested by the Blaine method of GB 175-2007 [29], and the results are shown in Figure 3. The specific surface areas of CFA, RM, BFS, CC, FBFS, PS, and GS were 525m^2^/kg, 734 m^2^/kg, 446 m^2^/kg, 378 m^2^/kg, 449 m^2^/kg, 425 m^2^/kg and 400 m^2^/kg, respectively.

### 2.2. Experimental Design of CSM

According to the requirements of the GB/T 41060-2021 [30], CSM1, CSM2, and CSM3 were prepared by CFA, RM, BFS, CC, and a silicon-aluminum-based solid waste (FBFS/PS/GS), as shown in Table 2. The different mass ratios of (T-CaO)/(SiO_2_ + Al_2_O_3_) (Ca/(Si + Al)) of the three CSMs were also calculated. Table 2 shows that the mass ratios of Ca/(Si + Al) of CSM1, CSM2, and CSM3 are 0.79, 0.81 and 0.75, respectively. The comprehensive comparison of the frost resistance and microstructure of the three CSMs is analyzed in the next section based on different mass ratios of Ca/(Si + Al).

### 2.3. Technical Framework of CSM

**Figure 4:**
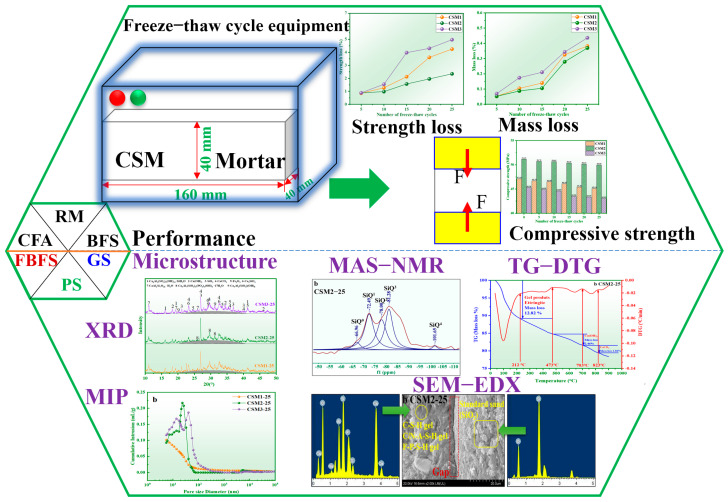
Technical framework of CSM.

The performance and microstructure items of CSMs are summarized in Figure 4. According to the dosage of the raw materials established in Table 2, CSM (40 × 40 × 160 mm^3^) was produced. The mortars were prepared by raw material (450 g) and standard sand (1350 g) with a mass ratio of 1:3 (15 mortar samples were prepared corresponding to each CSM). Then, the CSM mortar was stored for 28 days in a standard curing box with temperature (20 ± 1 °C) and relative humidity (95 ± 1%). At the same time, the strength value of the CSMs was evaluated on the press display before freeze–thaw cycling. The strength loss rate and mass loss rate of the CSM mortar hardened body were calculated according to GB/T 41060-2021 [30], as shown below.

The strength loss rate of CSMs was calculated according to Formula (1).

(1) $$S=\frac{e_{0}-e_{i}}{e_{0}}\times 100\%$$

*S*—Strength loss rate of CSM after *i* freeze–thaw cycles (%);
*e*_0_—Compressive strength value of CSM before freeze–thaw cycling (MPa);
*e_i_*—Compressive strength value of CSM after *i* freeze–thaw cycles (MPa).

The mass loss rate of CSMs was calculated according to Formula (2).

(2) $$\omega =\frac{m_{0}-m_{i}}{m_{0}}\times 100\%$$

*ω*—Mass loss rate of CSM after *i* freeze–thaw cycles (%);
*m*_0_—Mass value of CSM before freeze–thaw cycle (MPa);
*m_i_*—Mass value of CSM after *i* freeze–thaw cycles (MPa).

The frost resistance mechanism of CSM was analysed by XRD, MAS-NMR, TG-DTG, SEM-EDX, and MIP.

### 2.4. Test Methods

#### 2.4.1. Performance Test

According to the experimental operation of GB/T 41060-2021 [30], the compressive strength of the three CSMs was tested with standard press equipment (HYE-300-10). The mortar was put into the experimental box, and then the box with the CSMs was put into the freeze–thaw cycle equipment. The central temperatures of the CSMs during the freeze–thaw cycle experiment were −18 ± 2 °C and 5 ± 2 °C, respectively. A freeze–thaw cycle was completed within 7 h, and the melting and freezing time was not less than 3 h. The number of freeze–thaw cycles of the CSM mortar was 25 in this work, and the strength and mass values were tested by press equipment (HYE-300-10) and an electronic balance (JE3001) every 5 freeze–thaw cycles. The strength loss rate and mass loss rate of the CSM mortar hardened body were calculated by Formulas (1) and (2). Moreover, the mass percentage of f-CaO in CFA was accurately measured based on EN 451-1-2017 [31], and the SO_3_ content in CSM was determined by XRF. Then, the LOI of raw materials was obtained by a high-temperature furnace at 800 °C for 4 h according to GB 175-2007 [29], and the specific surface areas of raw materials were checked following GB 175-2007 [29].

#### 2.4.2. Microstructure Analysis

The mineral composition of CSMs was determined by a German D8 Advance X-ray diffractometer (XRD). The polymerization degree and structure of silicon aluminum in CSMs were tested by a JMM-EC600R ^29^Si MAS spectrometer. The mass loss of CSMs at different temperatures was detected by NETZSCH STA 449 F3/F5 instruments in the United States. The micromorphology of the CSMs and the interface between the paste and aggregate were photographed by a Gemini cold field scanning electron microscope and energy dispersive X-ray (SEM-EDX). The pore structure parameters of the CSMs were tested by AutoPore V 9620, a high-performance automatic mercury intrusion porosimeter (MIP) from the Mcmurrittick company in the United States. The pores of the CSM are filled with mercury under external pressure. The porosity can be calculated by data processing of the electrical signal generated by Mercury entering the CSM pores. Inductively coupled plasma–mass spectrometry (ICP–MS) 7800 (Agilent Corporation, Santa Clara, CA, USA) was used to analyze the leaching of Na, As, Cd, and Hg.

## 3. Results and Discussion

### 3.1. Strength Loss and Mass Loss

**Figure 5:**
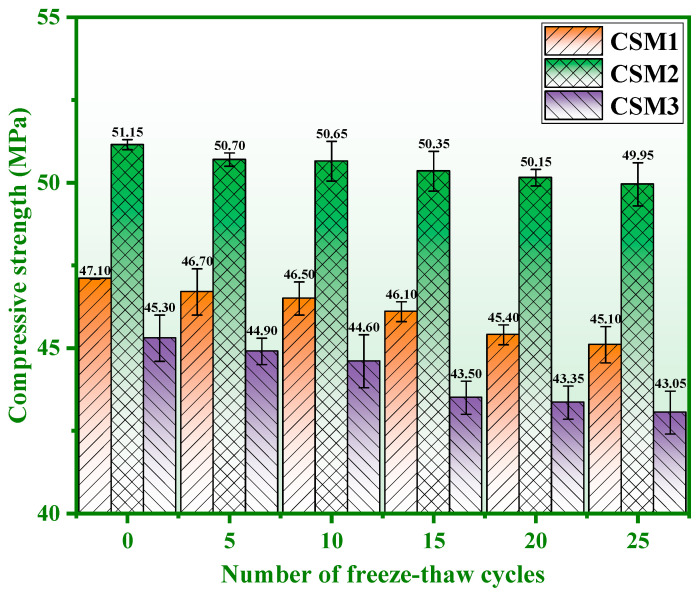
Strength of CSM at different freeze–thaw cycles.

**Figure 6:**
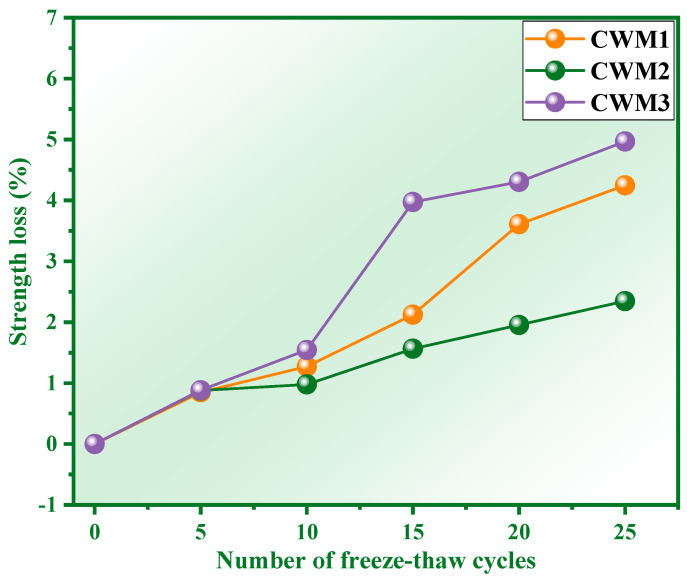
Strength loss rate of CSM at different freeze–thaw cycles.

The requirement for 42.5 fly ash Portland cement (P. F 42.5) based on GB 175-2007 [29] is that the compressive strength of the cementitious material is not less than 42.50 MPa after 25 freeze–thaw cycles (the compressive strength of CSMs is ≥15 MPa and ≥42.5 MPa at 3 days and 28 days, respectively). The compressive strength of CSMs with 0–25 freeze–thaw cycles is shown in Figure 5. It is obvious from Figure 5 that the compressive strength of the three CSMs gradually decreases with the number of freeze–thaw cycles from 0 to 25. This indicates that the compressive strength of the three CSMs is lost under the action of freeze–thaw cycles. The strength loss rate of CSMs corresponding to Figure 5 is shown in Figure 6. Figure 6 shows that the strength loss rate of the three CSMs gradually increases with the number of freeze–thaw cycles. Concretely, the strength loss rates of CSM1, CSM2 and CSM3 are 4.25%, 2.35% and 4.97% when the number of freeze–thaw cycles is 25, which meets the requirements (≤25.00%) of the strength loss rates in GB/T 41060-2021 [30]. In addition, the compressive strength values of CSM1, CSM2, and CSM3 are 45.10 MPa, 49.95 MPa and 43.05 MPa after 25 freeze–thaw cycles, which meet the strength requirements of P. F 42.5 in GB 175-2007 [29]. According to the results of Figure 5 and Figure 6, the compressive strength of CSM2 is higher than other CSMs and the strength loss rate of CSM2 is lower than other CSMs when the Ca/(Si + Al) mass ratio is 0.81. This means that the long-time frost resistance of CSM2 is optimal based on the synergy of CFA, BFS, RM, and PS.

**Figure 7:**
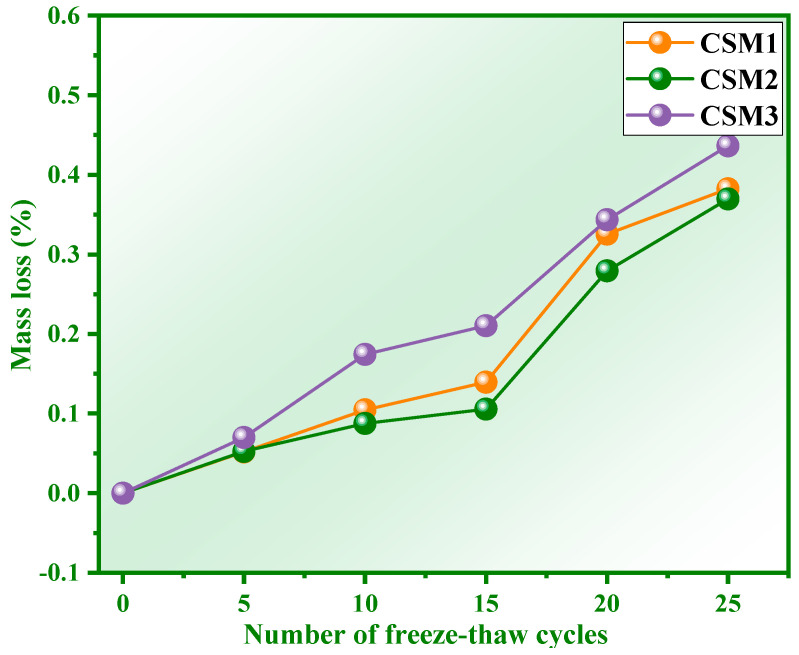
Mass loss rate of CSM at different freeze–thaw cycles.

Figure 7 shows the mass loss of the three CSMs at different numbers of freeze–thaw cycles. It is obvious from Figure 7 that the mass loss rate of the three CSM samples gradually increases with the number of freeze–thaw cycles from 0 to 25. This indicates that the tiny blocks are shed in the CSM matrix with the progress of the freeze–thaw cycle. The mass loss rates of the three CSMs are 0.38%, 0.36% and 0.44% when the number of freeze–thaw cycles is 25, which meets the requirements (≤5.00%) of the mass loss rate in GB/T 41060-2021 [30]. The mass loss rate of CSM2 is lower than that of the other CSMs when the Ca/(Si + Al) mass ratio is 0.81. This phenomenon indicates that the polymerization degree of the CSM2 system is higher, which is conducive to the stable connection of aggregates [32]. The frost resistance of CSM2 is optimal according to the comprehensive strength loss and mass loss.

### 3.2. Phase Composition Analysis

**Figure 8:**
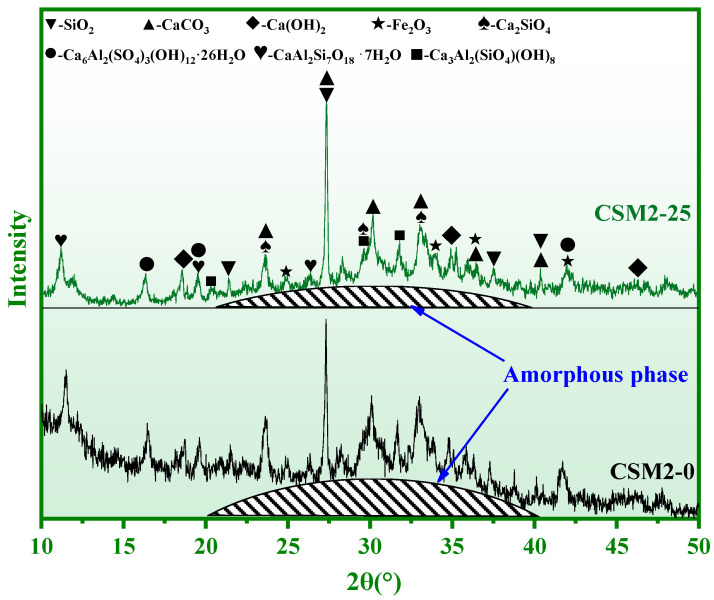
XRD results of CSM2 in 0 and 25 freeze–thaw cycles.

The phase composition of CSM2 after 0 and 25 freeze–thaw cycles (CSM2-0 and CSM2-25) is shown in Figure 8. The main phases of CSM2 are the amorphous phase, ettringite (Ca_6_Al_2_(SO_4_)_3_(OH)_12_·26H_2_O), portlandite (Ca(OH)_2_), calcite (CaCO_3_), metaheulandite (CaAl_2_Si_7_O_18_·7H_2_O), unreacted quartz (SiO_2_), hematite (Fe_2_O_3_), dicalcium silicate (Ca_2_SiO_4_), and katoite (Ca_3_Al_2_(SiO_4_)(OH)_8_). As shown in Figure 8, the phase type of CSM2 does not change with increasing freeze–thaw cycles. However, a comprehensive comparison of the XRD results shows that the diffraction peak intensity of the amorphous phase in CSM2 decreases slightly with the number of freeze–thaw cycles. This result indicates that a small number of amorphous phases in CSM2 were destroyed in the 25 freeze–thaw alternation environments.

**Figure 9:**
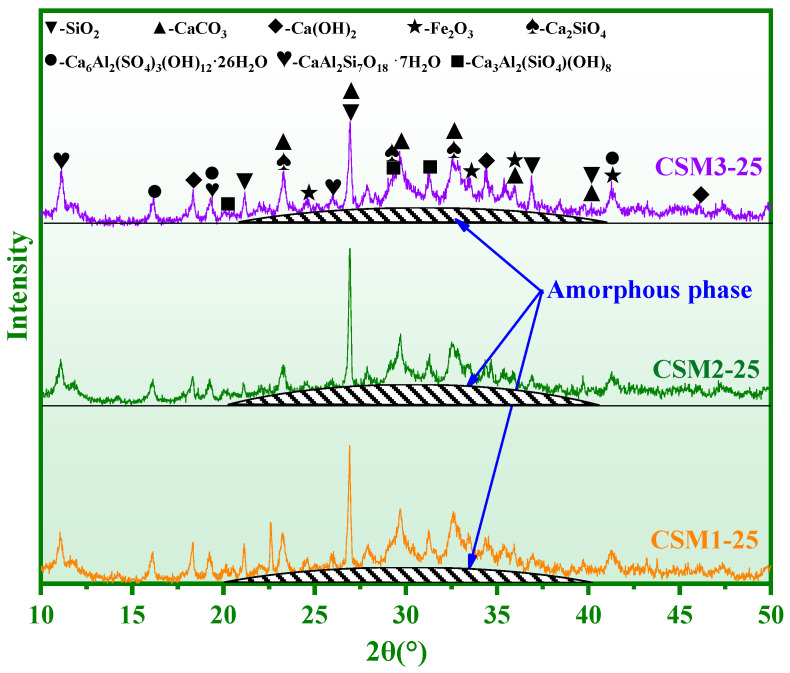
XRD results of three CSMs in 5 freeze–thaw cycles.

The phase composition of CSM1, CSM2, and CSM3 at 25 freeze–thaw cycles (CSM1-25, CSM2-25, and CSM3-25) is shown in Figure 9. According to the comprehensive comparison, the diffraction peak intensity of amorphous phases in CSM2 at 25 freeze–thaw cycles is higher than that of other CSMs when the mass ratio of Ca/(Si + Al) is 0.81. This result shows that the number of amorphous phases in CSM2-25 is the maximum based on the synergy of CFA, BFS, RM, and PS, which can improve the ability of CSM2 to resist freeze–thaw cycles. Therefore, the strength loss and mass loss of CSM2 is lower than those of the other CSMs after 25 freeze–thaw cycles.

### 3.3. Si-Al Structure Analysis

Nuclear magnetic resonance (NMR) spectroscopy is used to study the absorption of radio-frequency radiation by atomic nuclei, and it is the most powerful tool for qualitative analysis of the composition and structure of various inorganic substances. The number of relative bridge oxygen bonds in the ^29^Si NMR spectrum is represented by SiQ*^n^* (*n* = 0–4). Then, Zhang [33] found that the polymerization degree of silicon oxygen tetrahedral structure [SiO_4_] was quantitatively calculated by the number of relative bridge oxygen (RBO). The calculation formula of the polymerization degree of RBO in CSM is shown in Formula (3):

(3) $$\mathrm{RBO}=\frac{1}{4}(1\;\times \;\frac{Q^{1}}{\sum Q^{n}}+2\;\times \;\frac{Q^{2}}{\sum Q^{n}}+3\;\times \;\frac{Q^{3}}{\sum Q^{n}\;}+4\;\times \;\frac{Q^{4}}{\sum Q^{n}})=\frac{1}{4}\frac{\sum n\cdot Q^{n}}{\sum Q^{n}}$$

where Q*^n^* is the relative peak area of the ^29^Si NMR spectrum with RBO number *n*.

**Figure 10:**
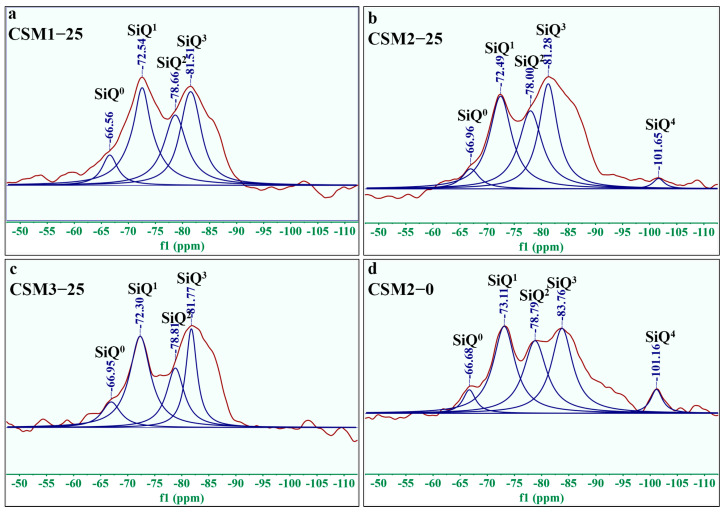
^29^Si NMR of three (**a**) CSM1-25, (**b**) CSM2-25, (**c**) CSM3-25, and (**d**) CSM2-0.

The ^29^Si NMR spectra and relevant data are shown in Figure 10 and Table 3, respectively. As seen from Figure 10 and Table 3, the [SiO_4_] of five relative bridge oxygen bonds are found in CSM: SiQ^0^, SiQ^1^, SiQ^2^(1Al), SiQ^3^(2Al) and SiQ^4^. Their relative peak areas were fitted by MestReNova software and are displayed in Table 3. Meanwhile, the degree of polymerization in the three CSMs was calculated according to Formula (3). SiQ^0^: Ca_2_SiO_4_ or Ca_3_SiO_5_ in CC; SiQ^2^ (1Al) or SiQ^3^ (2Al): C-A-S-H gel and N-A-S-H gel (C/N-A-S-H gel). In Table 3, SiQ^2^ or SiQ^3^ show a large peak area, which proves the presence of C/N-A-S-H gel in CSM. These results also indicate that the amorphous phase (Figure 8 and Figure 9) contains C/N-A-S-H gel, and this finding is consistent with that of Walkley et al. [34]. It can be seen from the ^29^Si NMR spectra of CSM2 in 0 and 25 freeze–thaw cycles (CSM2-0 and CSM2-25) in Figure 10b,d that there is a slight rise in the peak of SiQ^0^, and the peaks of SiQ^2^(1Al) (or SiQ^3^(2Al)) and SiQ^4^ become narrow when the number of freeze–thaw cycles for CSM2 is from 0 to 25. This phenomenon indicates that the C-S-H gel and C/N-A-S-H gel produced by CSM2 are slightly damaged under the action of freeze–thaw cycling. The ^29^Si NMR results of the three CSMs after 25 freeze–thaw cycles are comprehensively compared, as shown in Figure 10a–c. The SiQ^2^(1Al) (or SiQ^3^(2Al)) peak area of CSM is optimal (88.75 or 95.00) when the mass ratio of Ca/(Si + Al) is 0.81. Hence, the number of C/N-A-S-H gels is the maximum in CSM2-25. In addition, CSM2-25 contains an extra SiQ^4^ relative to the other CSMs. Therefore, the number of hydration products in CSM2-25 is more than that of other CSMs.

The ^29^Si NMR spectra of CSM1-25, CSM2-25, CSM3-25, and CSM2-0 were further analyzed by MestReNova, and the results are summarized in Table 3. The polymerization degree of CSM2 decreases slightly with the number of freeze–thaw cycles. This indicates that part of the RBO of [SiO_4_] in CSM2 is slightly destroyed during freeze–thaw cycles. The polymerization degree of CSM2-25 is highest (50.65%) when the mass ratio of Ca/(Si + Al) is 0.81. This phenomenon occurs as the formation of C/N-A-S-H gel and C-S-H gel is promoted by the participation of PS in the hydration reaction of CSM2. In summary, the frost resistance of CSM2 is optimal as the aggregate of CSM2 is closely connected by the high polymerization degree in the freeze–thaw cycle. The results of ^29^Si MAS NMR analysis correspond to XRD in CSM.

### 3.4. Thermogravimetric Loss Analysis

**Figure 11:**
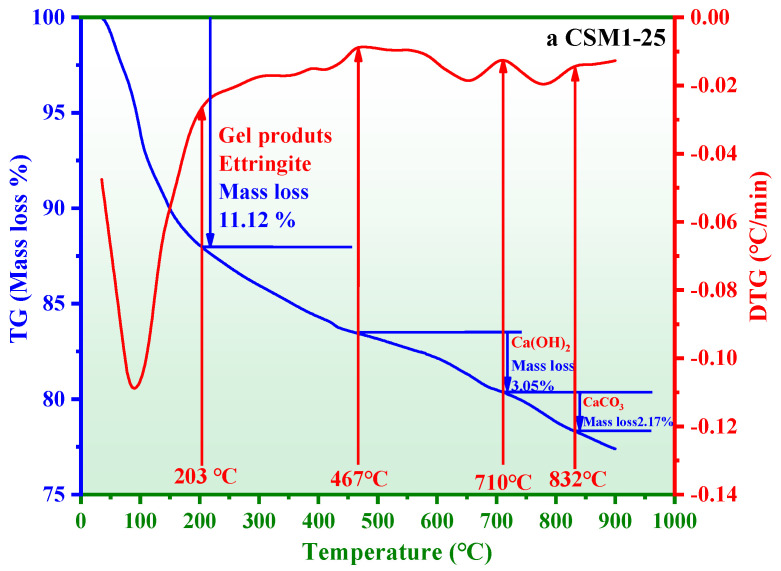

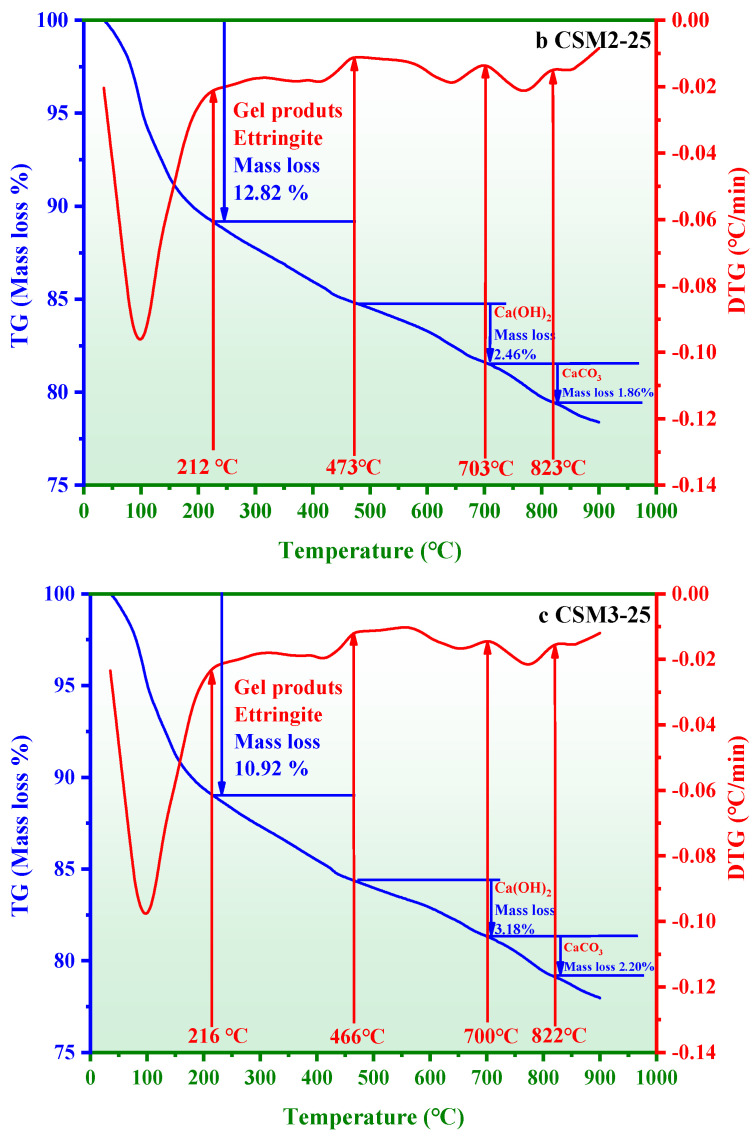
TG-DTG results of (**a**) CSM1-25, (**b**) CSM2-25, and (**c**) CSM3-25.

The thermogravimetric (TG) method is a technique used to measure the relationship between the mass loss and temperature under program-controlled temperatures. The differential thermogravimetric method (DTG) is the first derivative curve of the TG curve. The corresponding phase of mass loss at 60–216 °C is the thermal decomposition of gel products (C-S-H gels and C/N-A-S-H gels) and ettringite [35,36,37]. The corresponding phases of mass loss at 466–710 °C and 710–832 °C are attributed to the decomposition of Ca (OH)_2_ and CaCO_3_, respectively [38,39]. Figure 11 shows the TG-DTG results of CSM1, CSM2, and CSM3 after 25 freeze–thaw cycles (CSM1-25, CSM2-25, and CSM3-25). The mass losses of CSM1-25, CSM2-25, and CSM3-25 in the range of 60–216 °C are 11.12%, 12.82%, and 10.92%, respectively. This result shows that the relative quantities of C-S-H gels, C/N-A-S-H gels, and ettringite are greatest at CSM2-25 when the Ca/(Si + Al) mass ratio is 0.81. This phenomenon shows that many hydration products of CSM2 are generated based on the synergistic effect of CFA, BFS, RM, and PS, which is conducive to improving its frost resistance. The mass losses of CSM1-25, CSM2-25, and CSM3-25 at 466–710 °C are 3.05%, 2.46%, and 3.18%, respectively. This result suggests that the residual Ca(OH)_2_ after the hydration reaction is the minimum in CSM2, with a Ca/(Si + Al) mass ratio of 0.81. The mass losses of CSM1-25, CSM2-25, and CSM3-25 at 710–832 °C are 2.17%, 1.86%, and 2.20%, respectively. This phenomenon indicates that minimum carbonization of CSM2-25 occurs. The reason for the above results is that the formation of hydration products in CSM2-25 is more easily promoted under the action of PS to fill the pores compared with FBFS and GS. Therefore, the frost resistance of CSM2 is superior to those of CSM1 and CSM3. The TG-DTG results were consistent with the XRD and ^29^Si MAS NMR results.

### 3.5. Microstructure Analysis

**Figure 12:**
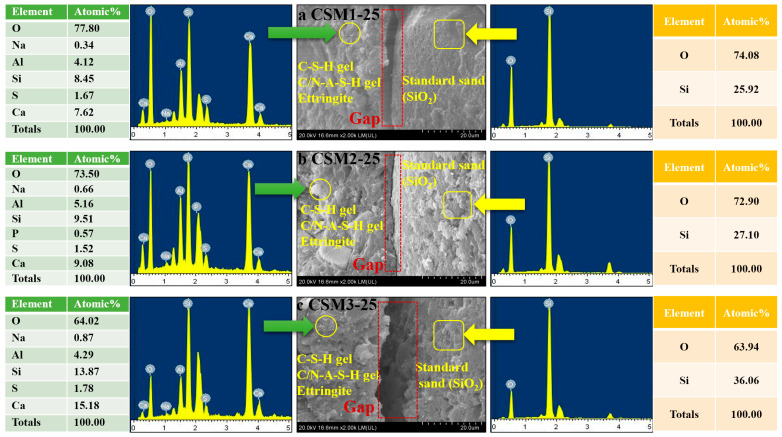
SEM-EDX results of (**a**) CSM1-25, (**b**) CSM2-25, and (**c**) CSM3-25.

The micromorphological characteristics of CSMs, the interface between paste and aggregate (standard sand), and the mineral distribution were obtained by SEM-EDX with a magnification of 2.0 k. The SEM-EDX of three CSMs after 25 freeze–thaw cycles (a CSM1-25, b CSM2-25, and c CSM3-25) is shown in Figure 12. From the SEM-EDX results in Figure 12a, the hydration products (C-S-H gel, C/N-A-S-H gel, and ettringite) are on the left and are distributed in layers with a small number of voids [19]. The interface between the pastes and standard sand (SiO_2_) is approximately 20.00 µm. The reason for this phenomenon is that the volume of water in the pores of CSM1-25 expands due to freezing, resulting in many cracks during the freeze–thaw cycle. Similarly, the left side in Figure 12c is the collection of flocculent C-S-H gel, C/N-A-S-H gel and acicular ettringite in CSM3-25, and the right side is standard sand (SiO_2_) [40,41]. The interface between the pastes and aggregates is accompanied by a gap of approximately 50.00 µm. This indicates that the internal structure of CSM3 is damaged in the freeze–thaw cycle. However, compared with Figure 12a,c, the micromorphology of Figure 12b is relatively dense, and the gap between the pastes and aggregates is less than 2.00 µm. This indicates that the connection of paste and aggregate in CSM2 is the greatest. It can be explained that the synergy enhancement of CFA, BFS, RM, and PS of CSM2 is optimal when the Ca/(Si + Al) mass ratio is 0.81, which forms many hydration products to improve the connection of paste and aggregate. The SEM-EDX results correspond to the XRD, ^29^Si NMR, and TG-DTG results.

### 3.6. Pore Structure Analysis

**Figure 13:**
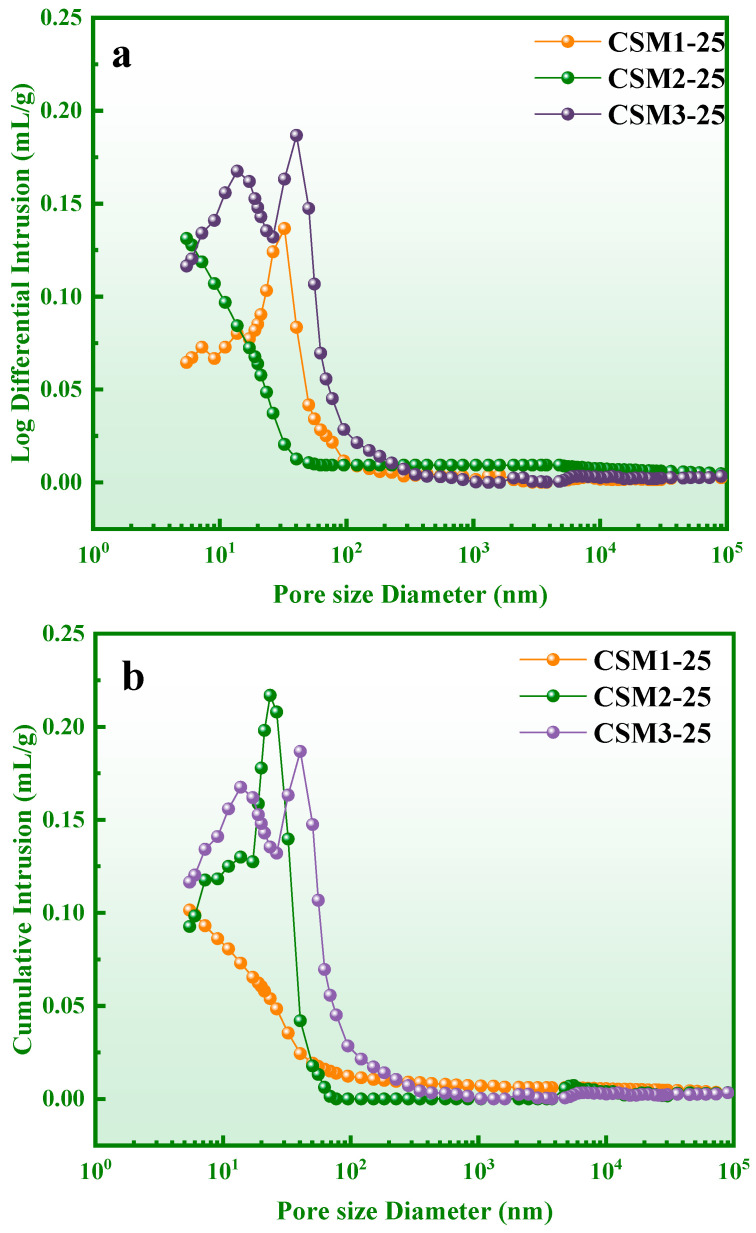
Log differential intrusion (**a**) and cumulative intrusion (**b**) of CSMs-25.

The pore parameter distribution is one of the main factors affecting the frost resistance of cementitious materials. The pore structure of the CSM pastes was determined by mercury intrusion porosimetry (MIP). As shown in Figure 13, the log differential intrusion and cumulative intrusion curves of CSMs after 25 freeze–thaw cycles (CSM1-25, CSM2-25, and CSM3-25), and pore structure parameters such as total pore volume, average pore diameter, porosity, and density are listed in Table 4. The total pore volume, average pore diameter, and porosity of CSM2-25 were 0.1312 mL/g, 15.1200 nm, and 23.14%, respectively. Although the total pore volume of CSM2-25 is higher than that of CSM1-25, the average pore parameter of CSM2-25 is lower than that of CSM1-25. Meanwhile, the bulk density and apparent density of CSM2-25 are 1.7636 and 2.2947 g/mL, respectively, which are higher than those of CSM1-25 and CSM3-25. This shows that the compactness of the pore structure in CSM2 is higher than those of other CSMs, which is conducive to reducing the formation of new cracks during the freeze–thaw cycle [42]. The reason for this phenomenon is that the synergistic effect of CFA, BFS, RM, and PS in CSM2 is optimal. Therefore, the strength loss and mass loss of CSM2 are lower than those of the other CSMs at the same number of freeze–thaw cycles.

**Figure 14:**
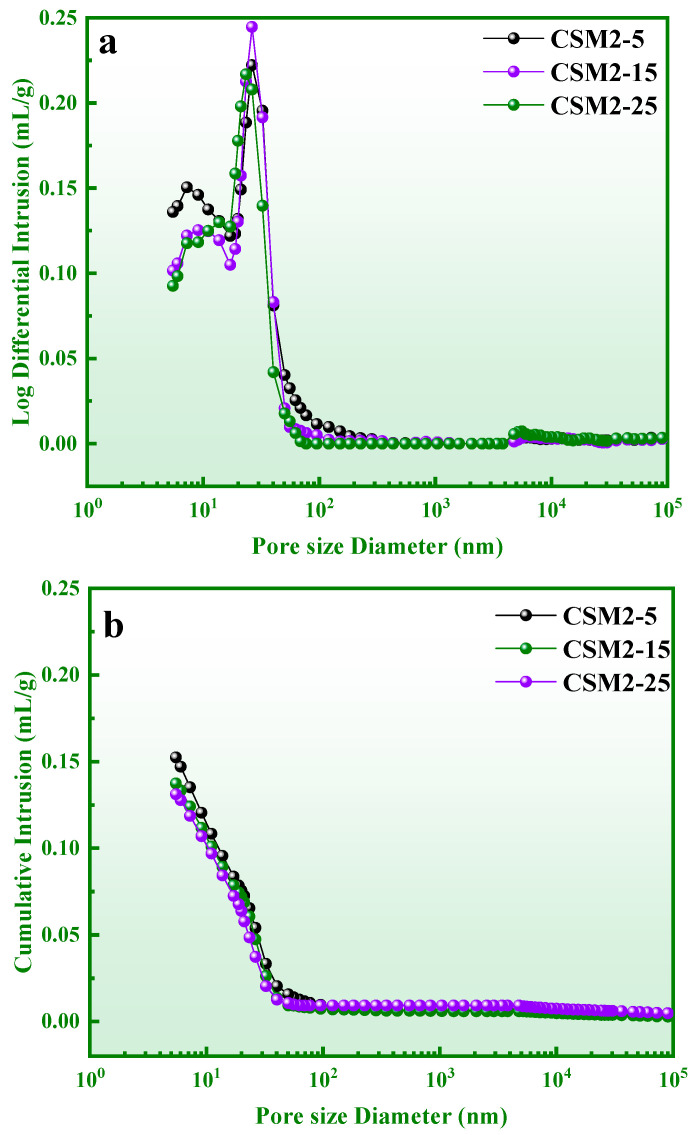
Log differential intrusion (**a**) and cumulative intrusion (**b**) of CSM2-5~25.

As shown in Figure 14, the log differential intrusion and cumulative intrusion curves of CSM2 at five, 15, and 25 freeze–thaw cycles (CSM2-5, CSM2-15, and CSM2-25), and pore parameters are shown in Table 5. According to the results in Figure 14 and Table 5, the total pore volume and porosity of CSM2 slightly decrease, while the bulk density and apparent density gradually increase with the number of freeze–thaw cycles from 0 to 25. This result indicates that CSM2 can still maintain a compact structure to reduce crack generation during freeze–thaw cycles. The reason for this phenomenon is that a better pore structure is promoted, based on the high degree of polymerization and the dense microstructure. Thus, the strength and mass of CSM2 are maintained by the better pore structure during the freeze–thaw cycle.

### 3.7. Leaching of Harmful Elements

The premise of solid waste utilization is that the leaching of harmful elements must be qualified [43]. Therefore, it is important that the leaching of harmful elements in CSMs meet environmental requirements after 25 freeze–thaw cycles (CSMs-25) [44]. Leaching tests of three CSMs-25 are performed according to Chinese standard GB 5086.1-1997 in this research [45]. The liquid/solid ratio is 10, and the turnover frequency is 30 ± 2 r/min (18 h). Then, ICP–MS was used to detect the leaching results of Na, As, Cd, and Hg. Finally, the leaching results of CFA, RM, PS, GS, CSM1-25, CSM2-25, and CSM3-25 are shown in Table 6. It is obvious that the leaching results of Na, As, Cd, and Hg in CFA, RM, PS, and GS exceed the WHO standard for drinking water [46], and these results are unqualified. However, the leaching concentration of harmful elements in the three CSMs-25 meets the WHO requirements for safe drinking water [46]. As such, the leaching concentration of Na^+^ in CSM2 is lower than that of other CSMs when the mass ratio of Ca/(Si + Al) is 0.81. The leaching concentrations of Na, As, Cd, and Hg in CSM2-25 are 82.6927 mg/L, 0.0025 mg/L, 0.0005 mg/L, and <0.0001 mg/L. These findings indicate that the Na, As, Cd, and Hg of CFA, RM, PS, and GS are still consolidated by CSM2 at 25 freeze–thaw cycles. The reason for this phenomenon is that the consolidation capacity of CSM2 is optimal based on the synergy of CFA, BFS, RM, and PS during 25 freeze–thaw cycles. Thus, CSM2 is a green cementitious material with long-term frost resistance.

## 4. Conclusions

In this work, the strength loss, mass loss, and frost resistance mechanism of circulating fluidized bed fly ash (CFA)-based multisolid waste cementitious material (CSM) are discussed. The major conclusions are as follows.

(1) The strength loss, mass loss, and strength of CSM2 after 25 freeze–thaw cycles (CSM2-25) are 2.35%, 0.36%, and 49.95 MPa, respectively, which meets the performance requirements of fly ash Portland cement (42.5#). The frost resistance of CSM2 is excellent based on synergy of CFA, BFS, RM, and PS.
(2) The thermogravimetric loss and polymerization degree of hydration products in CSM2-25 are 12.82 wt.% and 50.65%, respectively, which are higher than those of other CSMs. The reason is that the amount of hydration products (C-S-H gel, C/N-A-S-H gel, and ettringite) in CSM2 is the maximum during the freeze–thaw cycle.
(3) The interface between paste and aggregate, micromorphology, and pore structure of CSM2 are the densest based on the degree of high polymerization. These characteristics are beneficial to the stable development of long-term frost resistance in CSM2.
(4) The leaching concentrations of Na, As, Cd, and Hg in CSM2-25 are 82.6927 mg/L, 0.0025 mg/L, 0.0005 mg/L, and <0.0001 mg/L, respectively, during the action of 25 freeze–thaw cycles, which still meet the WHO standard of drinking water. Therefore, CSM2 is a green cementitious material with long-term frost resistance.

## Figures and Tables

**Table 1:** Chemical composition of raw materials.

Oxide	T-CaO	f-CaO	SiO_2_	Al_2_O_3_	SO_3_	Fe_2_O_3_	MgO	TiO_2_	P_2_O_5_	F	K_2_O	Na_2_O	LOI	Total
CFA	12.73	4.10	34.15	24.04	6.67	5.31	1.25	0.76	0.23	-	0.82	0.20	10.38	96.54
RM	21.09	-	19.02	22.46	0.29	15.15	0.46	4.33	0.67	-	0.57	6.01	8.84	98.89
BFS	34.14	-	34.64	18.64	1.66	0.86	6.96	0.77	0.04	-	0.63	0.62	0.50	99.46
CC	63.87	-	22.75	5.76	0.38	3.17	2.06	0.24	0.19	-	0.82	0.33	0.03	99.60
FBFS	39.74	-	29.19	15.02	2.73	0.82	9.38	0.81	0.02	-	0.50	0.47	0.60	99.28
PS	46.67	-	36.79	2.93	1.30	0.13	1.32	0.20	3.34	3.04	0.69	0.98	1.85	99.24
GS	26.41	-	34.79	15.96	0.49	9.53	0.98	0.89	0.07	-	0.70	4.89	3.94	98.65

Note: The loss on ignition (LOI) of CFA, RM, BFS, FBFS, PS, GS, and CC was measured at 800 °C for 4 h.

**Table 2:** Proportion of raw materials and Ca/(Si + Al) mass ratio (wt.%).

Sample	CFA	RM	BFS	CC	Silicon-Aluminum-Based Solid Wastes	Ca/(Si + Al) Mass Ratio
CSM1	30	10	20	30	10 (FBFS)	0.79
CSM2	30	10	20	30	10 (PS)	0.81
CSM3	30	10	20	30	10 (GS)	0.75

**Table 3:** Relevant data of ^29^Si MAS NMR in CSM.

Sample	Peak Position (PPM)	Assign	Relative	Polymerization Degree of RBO
CSM1-25	−66.56	SiQ^0^	22.22	48.95%
	−72.54	SiQ^1^	100.00	
	−78.66	SiQ^2^ (1Al)	77.78	
	−81.51	SiQ^3^ (2Al)	88.89	
CSM2-25	−66.96	SiQ^0^	16.25	50.65%
	−72.49	SiQ^1^	100.00	
	−78.00	SiQ^2^ (1Al)	88.75	
	−81.28	SiQ^3^ (2Al)	95.00	
	−101.65	SiQ^4^	6.25	
CSM3-25	−65.95	SiQ^0^	25.49	44.91%
	−72.30	SiQ^1^	100.00	
	−78.81	SiQ^2^ (1Al)	54.90	
	−81.77	SiQ^3^ (2Al)	56.86	
CSM2-0	−66.68	SiQ^0^	12.96	52.16%
	−73.11	SiQ^1^	100.00	
	−78.79	SiQ^2^ (1Al)	88.89	
	−83.76	SiQ^3^ (2Al)	96.30	
	−101.16	SiQ^4^	14.81	

**Table 4:** Pore structure parameters of CSM1-25, CSM2-25, and CSM3-25.

Samples	Total Pore Volume (mL/g)	Average Pore Diameter (nm)	Porosity (%)	Bulk Density (g/mL)	Apparent Density (g/mL)
CSM1-25	0.1014	18.0600	17.59	1.7339	2.1040
CSM2-25	0.1312	15.1200	23.14	1.7636	2.2947
CSM3-25	0.1814	17.3200	27.1100	1.4941	2.0497

**Table 5:** Pore structure parameters of CSM2-5, CSM2-15, and CSM2-25.

Samples	Total Pore Volume (mL/g)	Average Pore Diameter (nm)	Porosity (%)	Bulk Density (g/mL)	Apparent Density (g/mL)
CSM2-5	0.1524	14.9700	23.84	1.5637	2.0531
CSM2-15	0.1374	15.3900	23.19	1.6881	2.1976
CSM2-25	0.1312	15.1200	23.14	1.7636	2.2947

**Table 6:** Leaching results of heavy metals (mg/L).

Sample (mg/L)	Na	As	Cd	Hg
CFA	5.2753	0.0441	0.0015	<0.0001
RM	685.6372	0.0491	0.0016	0.0022
PS	12.5648	0.0007	<0.0001	<0.0001
GS	37.3006	0.0046	0.0005	<0.0001
CSM1-25	96.3796	0.0025	0.0003	<0.0001
CSM2-25	82.6927	0.0025	0.0005	<0.0001
CSM3-25	105.5107	0.0012	0.0007	<0.0001
WHO drinking water standard [46]	≤200.0000	≤0.0100	≤0.0030	≤0.0010

## Data Availability

Data sharing is not applicable to this article.
